# Native American Admixture in the Quebec Founder Population

**DOI:** 10.1371/journal.pone.0065507

**Published:** 2013-06-12

**Authors:** Claudia Moreau, Jean-François Lefebvre, Michèle Jomphe, Claude Bhérer, Andres Ruiz-Linares, Hélène Vézina, Marie-Hélène Roy-Gagnon, Damian Labuda

**Affiliations:** 1 Centre de Recherche, CHU Sainte-Justine, Université de Montréal, Montréal, Québec, Canada; 2 Projet BALSAC, Université du Québec à Chicoutimi, Chicoutimi, Québec, Canada; 3 Department of Genetics, Evolution and Environment, University College London, London, United Kingdom; 4 Department of Epidemiology & Community Medicine, University of Ottawa, Ottawa, Ontario, Canada; 5 Département de Pédiatrie, Université de Montréal, Montréal, Québec, Canada; University of Utah, United States of America

## Abstract

For years, studies of founder populations and genetic isolates represented the mainstream of genetic mapping in the effort to target genetic defects causing Mendelian disorders. The genetic homogeneity of such populations as well as relatively homogeneous environmental exposures were also seen as primary advantages in studies of genetic susceptibility loci that underlie complex diseases. European colonization of the St-Lawrence Valley by a small number of settlers, mainly from France, resulted in a founder effect reflected by the appearance of a number of population-specific disease-causing mutations in Quebec. The purported genetic homogeneity of this population was recently challenged by genealogical and genetic analyses. We studied one of the contributing factors to genetic heterogeneity, early Native American admixture that was never investigated in this population before. Consistent admixture estimates, in the order of one per cent, were obtained from genome-wide autosomal data using the ADMIXTURE and HAPMIX software, as well as with the fastIBD software evaluating the degree of the identity-by-descent between Quebec individuals and Native American populations. These genomic results correlated well with the genealogical estimates. Correlations are imperfect most likely because of incomplete records of Native founders’ origin in genealogical data. Although the overall degree of admixture is modest, it contributed to the enrichment of the population diversity and to its demographic stratification. Because admixture greatly varies among regions of Quebec and among individuals, it could have significantly affected the homogeneity of the population, which is of importance in mapping studies, especially when rare genetic susceptibility variants are in play.

## Introduction

A major goal of medical and population genetics is to understand phenotypic consequences of genetic variation [1]. For years, studies of founder populations and genetic isolates represented the mainstream of the genetic mapping effort in targeting rare single gene defects held to cause Mendelian disorders [2]. Searching for genetic determinants of complex disorders changed the focus from rare deleterious mutations to susceptibility variants of common frequencies [3] and shifted attention towards association studies requiring very large and diversified cohorts. However, the importance of rare variants in genetic susceptibility to common diseases has been vindicated [4]. This paradigm change, from the causal common to causal rare susceptibility variants, has renewed interest in founder populations [5]. In populations arising from a founder event or in a genetic isolate, an initially “rare” mutation may gain in frequency to become more “mappable” [6]–[9].

Numerous founder events accompanied European colonization of the Americas, creating populations that remained isolated due to geographic barriers and/or distinctive cultural/religious/ethnic identities [10]–[13]. Their putative cultural and genetic homogeneity (enhanced by demographic bottlenecks typical of New World settlements) are considered as important advantages in association studies [14]. But is this really so? The population descending from settlers of *Nouvelle-France*, of European and mainly French origins, forming the majority of today’s province of Quebec, Canada, is known for a number of recessive diseases that are endemic, of increased frequency and/or due to a population specific mutation(s) [15], [16]. Because of its relatively limited number of European founders, the genetic homogeneity of French Canadians has been implicitly assumed, due to a founder effect reinforced by a demographic spurt in the 19^th^ century [17], [18]. However, non-disease oriented genealogical and genetic studies have shown that the population of Quebec is more genetically diversified than previously anticipated [19], [20]. This can be ascribed to highly variable genetic contribution of distinct founders and to uneven geographic expansion of their descendants within the new colony. While designing association studies, one must be aware of genetic stratification within the patient and the corresponding control group, reflecting demographic history of the sampled population. Therefore, there is a need to understand the relative effects of demographic and genetic forces on the apportionment of genomic diversity among individuals and populations, and to be able to distinguish ancient ancestral relations from more recent admixture [21].

In addition to a diverse contribution of European founders [22], including a minute African origin [23], the resulting population of today’s Quebec was also genetically enriched by Native American admixture [19]. Indeed, the presence of Native Americans among the founders of *Nouvelle-France* is documented in historical records [24]–[26]. Their contribution was revealed by genetic studies of uniparentally transmitted markers showing the existence of Native American mitochondrial DNA lineages in the contemporary Quebec population [27], [28]. However, the extent of Native American contribution to the genetic makeup of the contemporary Quebec population is largely unknown. Genetic information limited to uniparentally transmitted Native lineages, and especially maternal lineages, is insufficient to quantify the extent of nuclear DNA admixture [28], [29]. Native ancestry is underreported in genealogical records although the extent of missing information remains an unsettled issue [26]. On the other hand, for a given individual, full Native ancestry is assumed once it is recorded, so that if this is not truly the case, it may skew our genealogical estimates of the Native genetic contribution. In genetic epidemiological surveys, the genetic homogeneity of the Quebec founder population was often taken for granted. However, assuming no admixture when in fact there is one, may lead to erroneous results in genetic mapping and association studies. In light of our results, the Quebec population is not different from other New World populations of European descent in being enriched in alleles of Native American origin, although the extent of admixture is much lower than in Central and South American populations [21], [30]–[32]. Similarly as well, the admixture was mainly through marriages with Native women [27], [28].

**Figure 1:**
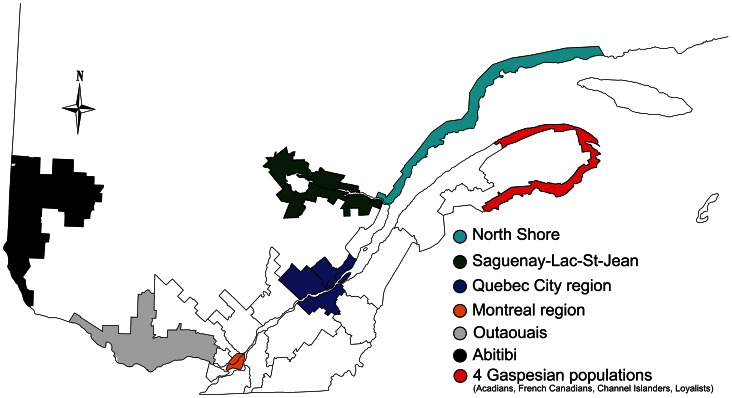
Map of Quebec subpopulations. In colors are the 10 regions/subpopulations included in the analyses.

The aim of the present study was to obtain a more reliable estimate of the Native American ancestry in the Quebec genome using autosomal DNA diversity as well as genealogical data. This study builds on single nucleotide variations (SNVs) from 205 individuals representing different regional groups of the contemporary Quebec population (Figure 1), some of which were previously analysed in a different context [20], [28]. For all but ten of these individuals (n = 195; Table 1), the ascending genealogies were reconstructed up to the Quebec founders. The genomic data of our reference populations were from Reich et al. [33].

**Table 1:** Native American ancestry proportions in the Quebec regions.

Regional/ethnocultural population group	Expected genetic contributionof Native Americans(genealogical data)		Native American ancestry (ADMIXTURE)		Native American SNVs (HAPMIX)		Total length of haplotypes shared with Native Americans (fastIBD)	
(n genotypes; n genealogies)	Mean (%)	SD	Mean (%)	SD	Mean (%)	SD	Mean (cM)	SD
Abitibi (18;18)	0.18	0.42	1.33	1.11	0.84	0.47	12	9
Outaouais (15;14)	0.24	0.43	1.94	1.43	1.19	0.97	24	32
Montreal (25;20)	0.12	0.13	1.76	0.97	1.09	0.57	16	8
Quebec City (25;22)	0.16	0.22	1.78	0.99	0.96	0.63	14	7
Saguenay (22;21)	0.17	0.18	1.95	1.69	1.41	1.73	27	62
North Shore (20;20)	0.42	0.57	2.42	1.51	2.05	1.64	47	47
Gaspesian Channel Islanders (20;20)	0.82	0.98	2.77	1.38	1.38	1.09	33	27
Gaspesian Acadians (20;20)	0.10	0.15	1.77	0.88	1.06	0.82	19	19
Gaspesian Loyalists (20;20)	0.14	0.31	2.70	1.16	0.87	0.48	16	15
Gaspesian French Canadians (20;20)	1.14	1.56	2.81	2.22	2.50	2.57	54	64
Quebec total sample (205;195)	0.35	0.73	2.12	1.43	1.33	1.34	26	37

Here Quebec regional/ethnocultural groups are presented with (number of individuals with genotype data; number of individuals with genealogical data). For each population, expected Native American genetic contribution was estimated using genealogical data. The Native American ancestry proportions in the Quebec subpopulations was estimated with 1) the ADMIXTURE software [43] 2) the HAPMIX software [39], and 3) IBD sharing analysis using the fastIBD software [41]. For each measure, we tested for differences among subpopulations using a Kruskal-Wallis test (Table S3).

## Materials and Methods

### Population Sample and Ethics Statements

We analyzed 205 unrelated (up to the 3^rd^ generation) individuals from 10 groups from Quebec (see map Fig. 1 and Table S1). Five regions were described in [20]: North Shore, Saguenay, and the areas of Quebec City and Montreal, as well as 3 Gaspesian ethnocultural groups (French Canadians, Loyalists and Acadians). In this study, we added 3 and 9 samples from the Montreal and Quebec City areas, respectively, in addition to samples from 3 new regions: Abitibi (18 samples), Outaouais (15 samples) and the Gaspesian-Channel Islander subpopulation (20 samples). All participants provided informed written consent, and the study was approved by the CHU Sainte-Justine Ethics Committee. Regional/ethnic affiliation was self-described by the participants. DNA was obtained for all participants as previously described [20], [34] and sent to the McGill University and Genome Quebec Innovation Center to be genotyped on Illumina HumanHap650Y and 610Quad arrays according to the recommended protocols. Quality control filters were applied at the individual and Single Nucleotide Variation (SNV) levels using the PLINK software v1.07 [35], [36] following the same criteria than in Roy-Gagnon et al. [20]. Information was collected to reconstruct the genealogy of each participant. Genealogies were reconstructed as far back as possible in a total of 195 individuals (genealogical data were missing for 5 individuals from the Montreal area, 3 from the Quebec City area, and 1 from both Outaouais and Saguenay) using the BALSAC population register [37] and the Early Quebec Population Register [38].

Unless stated otherwise, the Native American reference sample (n = 52) includes individuals from Northern America (Aleutians, Algonquin speakers, Chipewyans, Cree, Ojibwa as well as West and East Greenlanders), whereas the European reference sample (CEU+French) consisted of HapMap CEU (n = 108) and French (n = 28) from the Human Genome Diversity Panel (HGDP). Extended reference sample of Europeans contained in addition, HGDP Italians (n = 12) and Tuscans (n = 8), as well as HapMap Tuscans TSI (n = 88) (Table S2).

### Statistical Analysis

To infer local ancestry under a haplotype-based model, we used the HAPMIX software version 1.2 [39], [40] that estimates, at each locus, the probability of having 0, 1 or 2 alleles transmitted by one of the 2 source populations. We used an approach similar to Reich [33] for masking the European and African segments in the Native Americans masked dataset. We retained all loci with ≥0.95 probability that 1 or both alleles originated from the Native American source population. For this analysis we selected subsets of 50 individuals to match the sample size of the Native North American populations. Native American and European populations were phased together using the BEAGLE software version 3.3.2 [41], [42].

We estimated the global ancestry using the model-based approach implemented in the ADMIXTURE software version 1.22 [43], [44] with K = 3 (K being the number of ancestral populations assumed in the model) to distinguish between Native North American and European and Siberian ancestry in the Quebec sample. K = 3 allowed us to distinguish between old and new Asian-Native American ancestry. We used the PLINK software to select single nucleotide variations (SNVs) in approximate linkage equilibrium (pairwise r^2^<0.1 in sliding windows of size 50 shifting every 10 SNVs), yielding a subset of 46,344 SNVs.

We used fastIBD from the BEAGLE software version 3.3.2 [41] to find shared Identity-by-Descent (IBD) segments between individuals of Quebec and the Native Americans to investigate shared ancestry [45]–[47]. The fastIBD method is based on estimating frequencies of shared haplotypes allowing for phase uncertainty. Results from this method were shown to be well correlated with genealogical kinship coefficients in Quebec individuals. Following the authors’ recommendation, we performed 10 runs of fastIBD (*ibscale* = 4) that we merged using the scripts provided by the authors. We performed the analysis with the unmasked complete data and for each Native American individual separately, we discarded afterwards shared IBD segments located in masked genomic regions. We retained the remaining shared IBD segments of at least 1 cM. All IBD segments shared with Native Americans were finally pooled for each Quebec individual.

We calculated the *f_3_* statistic implemented in the ADMIXtools software [40], [48], which is based on patterns of allele frequency correlations across populations, to estimate the Native American ancestry proportion lower and upper bounds in the Quebec sample. The proportion of Native American ancestry in the Quebec sample was also evaluates using the ALDER software version 1.0 [49], [50]. Both ALDER and ADMIXtools (*rolloff* analysis) were used to estimate the linkage disequilibrium (LD) decay due to admixture. ALDER decay curves obtained using only one reference population were fitted starting at 0.8 and 0.7 cM (determined by the software) for the Quebec and French+CEU sample respectively using the masked Native North Americans as reference. The *rolloff* decay curve for the Quebec sample was estimated with two reference populations, Native North Americans (unmasked) and Europeans, and was fitted starting at 0.5 cM.

The R statistical environment version 2.15.0 Patched [51] was used for additional statistical testing and graphing.

### Genealogical Analysis

Of the 8424 founders (individuals whose parents could not be traced in Quebec parish and civil records) identified in the 195 genealogies of the Quebec individuals, 39 were of documented Native American origin. The genetic contribution (GC), based on genealogical data, is the expected proportion of the genome transmitted by an ancestor to a given individual. The GC was calculated using the GENLIB package, a genealogical analysis tool developed at BALSAC for the S-PLUS environment and transferred to the R environment for internal use. The mean GC was obtained by summing the GC of all Native American founders to all Quebec individuals and dividing by the number of individuals.

## Results

### Admixture Proportions Inferred using Genomic Data

**Figure 2:**
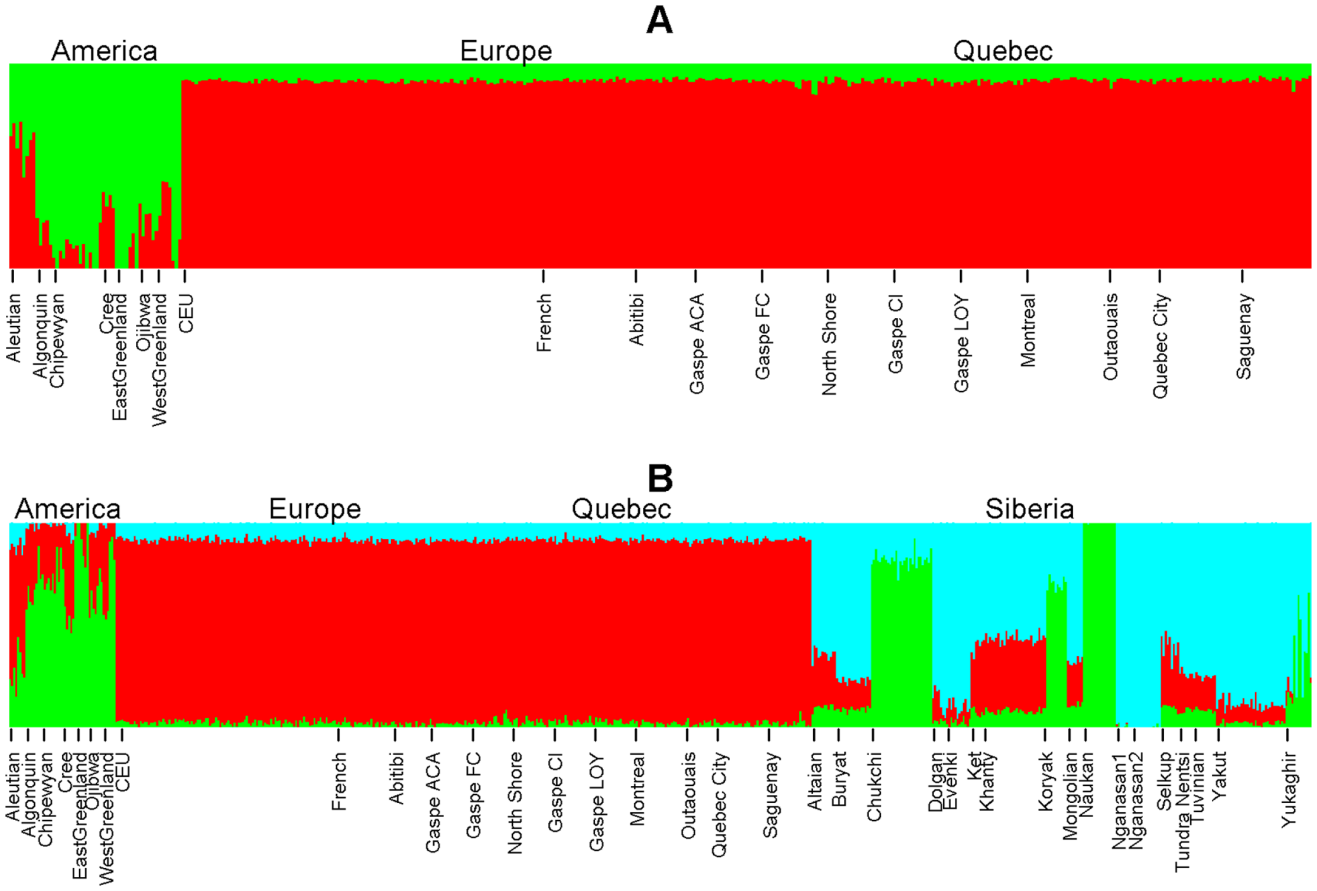
Barplots of ancestry proportions. The global ancestry was estimated using the model-based approach implemented in the ADMIXTURE software [43] : in Awith K = 2 to distinguish between Native American (green) and European (red) ancestry and in B with K = 3 in the presence of Siberian populations (cyan).

**Figure 3:**
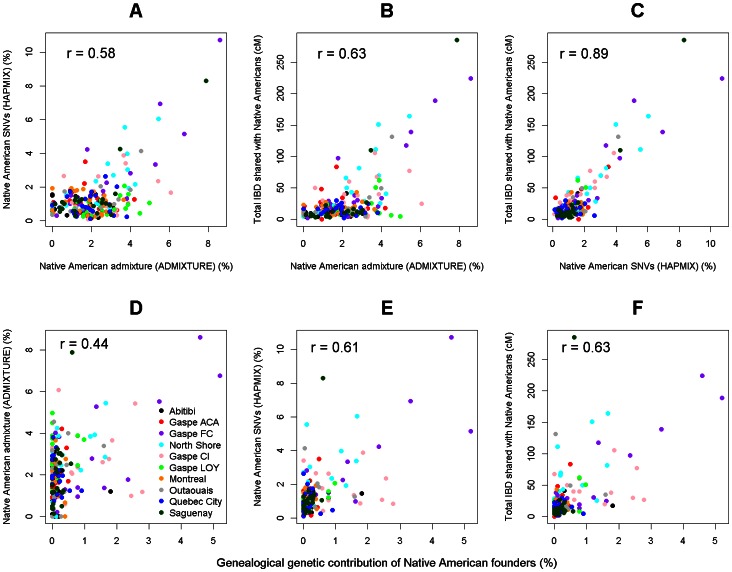
Scatter plots of correlations. Scatter plots showing the correlations between different Native American genetic ancestry estimates in the Quebec subpopulations (upper) and between genetic ancestry estimates and genealogical genetic contribution of Native American founders to the Quebec individuals (lower). The Pearson correlation coefficient (r) is shown on each plot.

Using HAPMIX [39], the overall degree of Native American admixture in the Quebec population was estimated at 1.3±1.3%, with 3-fold differences (0.84 to 2.50%) between population groups (Table 1) and much greater variation among individual genomes (Table S1). The results obtained by ADMIXTURE [43] were less straightforward. While this analysis captured differences in the extent of Native admixture among Quebec individuals (see Figures 2 and 3, and Table S1), it also detected some “background admixture” reflecting ancient common ancestry of Europeans and Native Americans, prior to European colonisation of the New World and presumably due to an earlier gene-flow between Eurasian populations [52] (Figure 2). In a simple K = 2 analysis (i.e., assuming two ancestral populations), we obtained an estimate of about 8% of Native American admixture both in Europe and in Quebec (Figure 2a). Adding Siberian populations in the K = 3 analysis significantly reduced this background effect, but the estimated degree of Native American admixture in Quebec, of 2.1±1.4%, still remained above that evaluated by HAPMIX and did not exceed the overall estimate in our European reference sample (2.3±1.2%). Nevertheless, in the ADMIXTURE graph it is easy to remark higher level of Amerindian admixture in some Quebec individuals, which appears as a signature of recent admixture unseen in European samples.

In this paper we also explore the use of Identity-by-Descent (IBD) estimation by fastIBD [41] to evaluate the extent of sharing of genomic segments between the Native genomes and Quebec individuals of primarily European descent. Of note, in this new application of fastIBD, rather than comparing individual Quebec and Native genomes one by one in a pairwise fashion, we analysed individual Quebec genomes against the entire set of Native North American samples. Based on this approach, the mean haplotype sharing in the whole Quebec sample was evaluated at 26±37 cM, roughly corresponding to about 0.8% of the genome, varying from 12 to 54 cM among different Quebec population groups (Table 1).

Importantly, estimates of Native American ancestry by the three methods that use genomic data are in very good agreement (Figure 3a, b and c). Discrepancies appear to be mainly due to individuals with a very low level of admixture where the signal-to-noise ratio is obviously very low. In the case of the estimates by ADMIXTURE, the effect of stochastic noise is additionally exacerbated by the genetic background mentioned above, which is much smaller but still present in the K = 3 analysis including Siberian samples (Figure 2b). This is presumably the reason why the best correlation is obtained between the fastIBD and HAPMIX estimates (Figure 3c), techniques that appear to be less sensitive to ancient admixture events and/or ancient common ancestry.

### Admixture Proportions Inferred Using Genealogical Data

When compared to genealogical estimates of the expected genomic contribution of Native American founders (Table S1), the best correlations were again obtained with HAPMIX and fastIBD results (Figure 3e and f; Pearson correlation coefficient r = 0.61 and 0.63, respectively). At the same time, however, we observed a fraction of individuals who display relatively low admixture in genealogical analysis but whose relatively high level of admixture was consistently detected in their genomic data (Figure 3). This is consistent with the hypothesis that genealogical information about the Native American origin of ancestors is likely incomplete, because it was either not recorded by the priest in charge of the parish registers or unknown for various reasons such as adoption or illegitimate birth. The average Native American contribution, estimated at 0.35% from the genealogical records, is thus expected to be an underestimate. Therefore, we also anticipate that having more complete genealogical information about Native American contributors would substantially improve the correlation between genomic and genealogical estimates of admixture (shown in Figure 3e and f).

### Time Since Admixture

**Figure 4:**
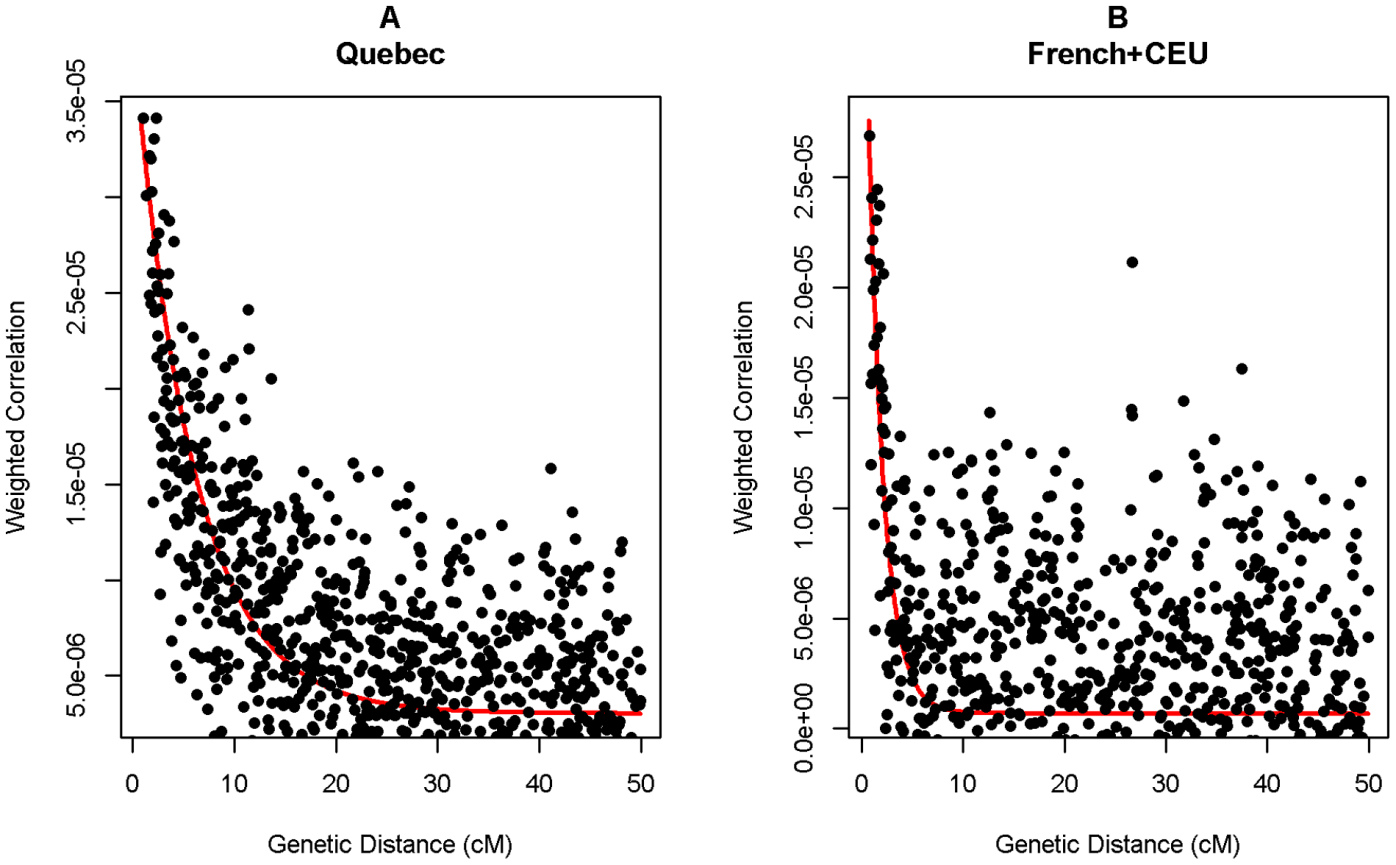
Plots of LD decay. LD decay of admixed fragments was assessed using the ALDER software [49] to test for admixture in the Quebec (A) and European (B) sample using the Native North Americans as the reference population.

Using the genealogical record, we can evaluate the time period of admixture. It extends from the beginning of the colony in the early 1600s to the first half of 19^th^ century (Figure S4), or roughly 7 to 13 generations ago. Note that Quebec individuals participating in this study and representing regional and/or ethnocultural groups of the Quebec population were unaware of and/or did not declare their admixed Native American ancestry. However, descendants of more recent mixed marriages considering themselves as Métis were not studied here. The time of admixture can be also estimated from the genomic data, from the decay of linkage disequilibrium around the reference, mutated or admixed, segment [9]. Such analysis to estimate time as well as the extent of admixture is implemented in the ADMIXtools software [48] and its recent avatar ALDER [49]. The *f_3_* statistic estimates of the proportion of admixture were between 0.5 and 2.9%, and the admixture time inferred by *rolloff* was about 13.6±2.5 generations ago (Table 2), i.e. about 435±80 years ago, assuming 32 years per generation [53]. Using only Native North American as a reference, ALDER estimated the lower bound of the proportion of admixture in Quebec at 0.8±0.2% and the admixture generation at 11.2±3.2, or 358±102 years ago (Figure 4a). The same estimates for the European sample were 1.0±0.3% and 1890±1600 years ago, respectively (Figure 4b). This is consistent with much older, yet not well characterized admixture events in Eurasia, such as, for example, the prehistoric gene-flow from Siberia to Northern-Europe [52]. Importantly, the above estimates of average time of admixture in Quebec, pointing to the turn of 17^th^ century, are in excellent agreement with historical data and the genealogical records (Figure S4), especially given the possibility of a slight upward bias in *rolloff* time estimates when the degree of admixture is low (<5%) [54].

**Table 2:** Native American ancestry proportions and age of admixture in the Quebec population sample by different methods and using different reference populations (see Table S2).

		Reference populations^a^		
		Native Americans,Europeans	Native North Americans, French+CEU	Unadmixed Native Americans, French+CEU
**Native American ancestry**	**Mean (%)**	3.24	2.12	4.32
**(ADMIXTURE)** a	**SD**	1.13	1.43	1.05
**Native American SNVs**	**Mean (%)**	0.90	1.33	0.99
**(HAPMIX)** b	**SD**	0.75	1.34	0.61
**Length of IBD sharing with**	**Mean (cM)**	11	26	9
**Native Americans**	**SD**	19	37	13
**Admixture proportions**	**Lower bound (%)**	1.2	0.5	0.6
**(** ***f_3_*** ** statistic, ADMIXtools)**	**Upper bound (%)**	−1.8	2.9	3.1
**Admixture proportions**	**Lower bound (%)**	0.8	0.8	0.7
**(ALDER)**	**SD**	0.2	0.2	0.2
**LD decay**	**Mean (gen)**	16.4	13.6	15.6
**(** ***rolloff*** **, ADMIXtools)**	**SD**	3.1	2.5	2.9
**LD decay**	**Mean (gen)**	13.6	11.2	13.6
**(ALDER)**	**SD**	4.3	3.2	4.2

a For ADMIXTURE, Siberians were used as a third reference population,. whereas IBD sharing and ALDER, used only Native Americans as a single reference population.

b For HAPMIX, 50 samples were randomly selected from each of the reference populations to match the Native North American sample size.

Moreover, it is plausible that part of the “missing” genealogical information involves individuals who were admixed prior to their first registration in Quebec civil records. For instance, in a previous study [27], [28] we identified Acadian settlers, whose Native ancestry was only discovered once their descendants mitochondrial DNA was investigated.

## Discussion

The advantages of using genetically isolated, founder populations in gene mapping have often been discussed in the context of studies on the genetic bases of Mendelian disorders, as well as on genetic susceptibility genes of complex diseases [14], [55], [56]. Some of the anticipated advantages can be compromised by a hidden population structure due to local founder effects [6], [19], [20], [57] and/or to unrecognized admixture. We found a low level of admixture overall. However, the variance of admixture estimates among individuals is very large (Table S1 and Figures S1, S2 and S3). Admixture estimates also vary significantly between the studied subpopulations (Kruskal-Wallis p<0.001 for genealogical and IBD estimates and p<0.05 for HAPMIX and ADMIXTURE estimates, Table S3). The four groups that consistently show the highest Native ancestry estimates are Gaspesians (ethnocultural groups of French Canadian or Channel Islander origin) as well as French Canadians in the North Shore and Saguenay regions.

These results were obtained using the sample of 52 Native North American genomes described above. In order to test how the choice of the Native American or European reference individuals affected our results, we conducted the same analyses using different reference populations. We observed a small difference in the extent of admixture using different sets of reference genomes. These differences likely reflect geographic proximity and time depth of shared ancestry of the populations we could analyze (Table 2 and Figures S1, S2 and S3).

Our study also shows that the analysis of IBD sharing performed very well in the analysis of recent admixture, comparable or perhaps even better than HAPMIX. Therefore, IBD sharing can be used to assist population structure analysis in association studies. Others have also shown that the analysis of IBD sharing is a promising tool for reconstructing populations’ demographic history [45]–[47], [58]. Interestingly, principal components analysis (PCA), often used in admixture studies, was not useful here because the observed level of Native American admixture was insufficient to be revealed in PCA plots (Figure S5). In contrast, approaches based on LD and haplotype information used here appear sensitive enough to capture the subtle recent Native American ancestry latent in the Quebec population. Otherwise it would be difficult to discern between the genetic sharing due to common ancient population history and recent admixture (Table 2, Figure 4).

In conclusion, using dense genotypic data and deep-rooted genealogical data, we estimated the Native American ancestry in a population sample from Quebec, Canada. The Native American genetic contribution calculated using genealogical data was low. Unlike most studied admixed populations that have greater admixture proportions [30], [59]–[61], we estimated the part of genetic ancestry coming from Native Americans to Quebec regional samples at about 1%. An individual separated by *m* meioses from an ancestor is expected to share 2^−*m*^ of this ancestor’s genome, i.e. on average about 0.1%, or 3.3 cM, after 10 generations. However, the length (exponentially distributed) of a shared segment has a mean of 100cM/*m*, or about 10 cM after 10 generations, suggesting that some individuals carry fairly long shared fragments and others not at all, explaining the variance among individual genomes. One percent Native ancestry can be understood as if everybody shared a Native American ancestor 6–7 generations ago. Indeed, a recent study based on four Quebec regional populations indicates that between 53 and 78% of Quebecers have at least one Native American ancestor in their genealogy [24]. Because of the small size of the early Quebec population, the same ancestor often contributed through more than one line to the same contemporary genome, thus suggesting its average occurrence at more distant generations than 6 or 7. Obviously, in historical reality, this genetic contribution varied both in time and space, impacting on stratification of the population and uneven distribution of the rare variants. Hence, Native American ancestry likely played a role in a reduction in the homogeneity of the Quebec founder population. This should be taken into account in the context of mapping and association studies, especially when rare genetic variants are involved.

## Supporting Information

Figure S1 **HAPMIX results in the Quebec population groups using different reference populations.** Boxplots of the percentage of Native American ancestry from 3 runs of the ADMIXTURE software performed with different reference populations listed in the titles of the individual plots. See Table S2 for the description of the reference populations.
(TIFF)

Figure S2 **ADMIXTURE results in the Quebec population groups using different reference populations.** Boxplots of total length of IBD sharing between the Quebec individuals and different Native American reference populations from 3 runs of fastIBD. See Table S2 for the description of the Native American populations.
(TIFF)

Figure S3 **IBD sharing between the Quebec population groups and different Native American populations.** Boxplots of total length of IBD sharing between the Quebec individuals and different Native American reference populations from 3 runs of fastIBD. See Table S2 for the description of the Native American populations.
(TIFF)

Figure S4 **Plot of the number (bars) and genealogical genetic contribution (dots) of the Native American (red) and non Native American (blue) founders by marriage year in the genealogies of the Quebec individuals.**
(TIFF)

Figure S5 **First 4 eigenvectors of the Principal Component Analysis of the genomic data.** Note - Global ancestry was estimated by PCA on the genotypic data using the EIGENSOFT software version 3.0 [40], [62]. To remove the effect of LD on the PCA, we used the subset of pruned SNPs described above.
(TIFF)

Table S1 **Genetic contribution and admixture indices among Quebec individuals.**
(DOCX)

Table S2 **Reference populations used in the analyses.**
(DOCX)

Table S3 **Genetic contribution and admixture indices in the Quebec subpopulations.**
(DOCX)
