# Supplementary figures and images for: Native American Admixture in the Quebec Founder Population
Source: PLoS One. 2013 Jun 12;8(6):e65507. doi: 10.1371/journal.pone.0065507 (PMC3680396; doi:10.1371/journal.pone.0065507)

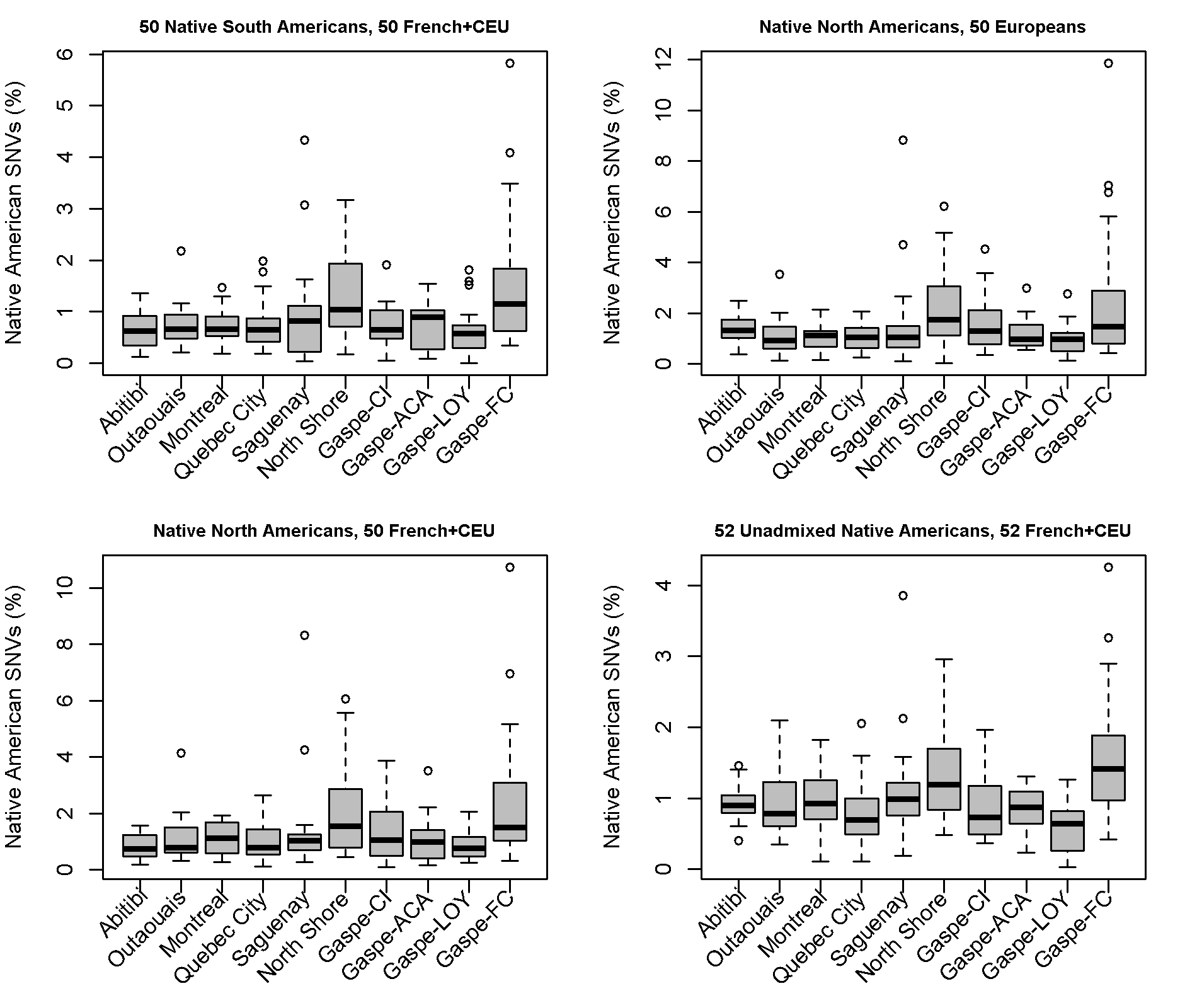

Supplement: Figure S1 — HAPMIX results in the Quebec population groups using different reference populations. Boxplots of the percentage of Native American ancestry from 3 runs of the ADMIXTURE software performed with different reference populations listed in the titles of the individual plots. See Table S2 for the description of the reference populations. (TIFF) [file pone.0065507.s001.tiff]

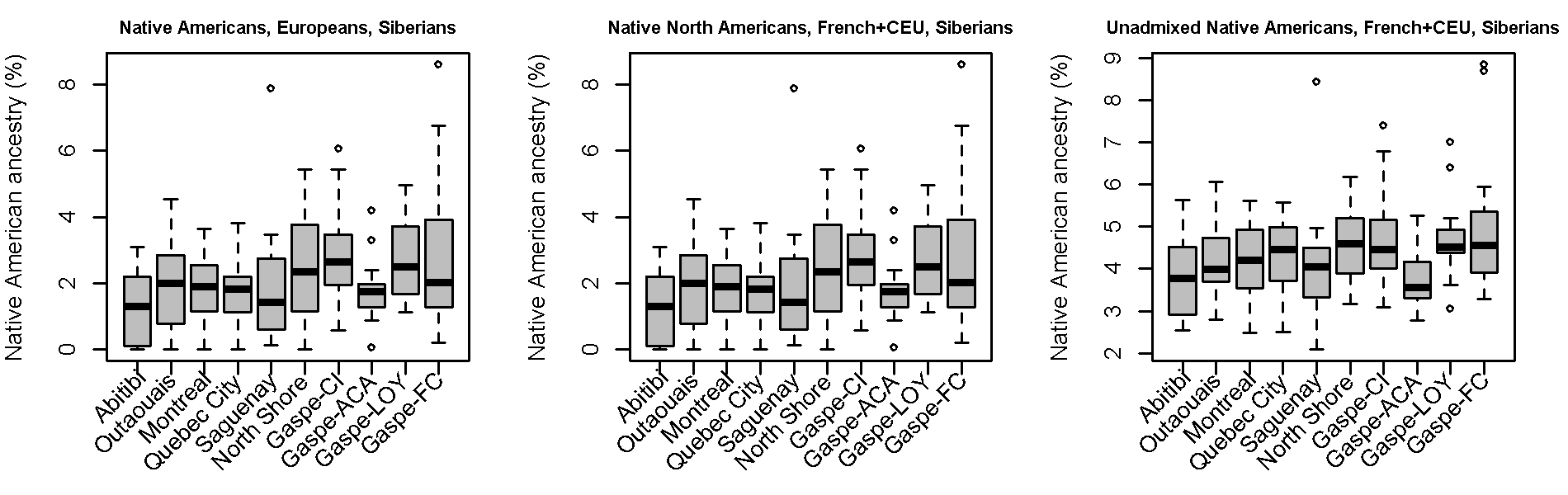

Supplement: Figure S2 — ADMIXTURE results in the Quebec population groups using different reference populations. Boxplots of total length of IBD sharing between the Quebec individuals and different Native American reference populations from 3 runs of fastIBD. See Table S2 for the description of the Native American populations. (TIFF) [file pone.0065507.s002.tiff]

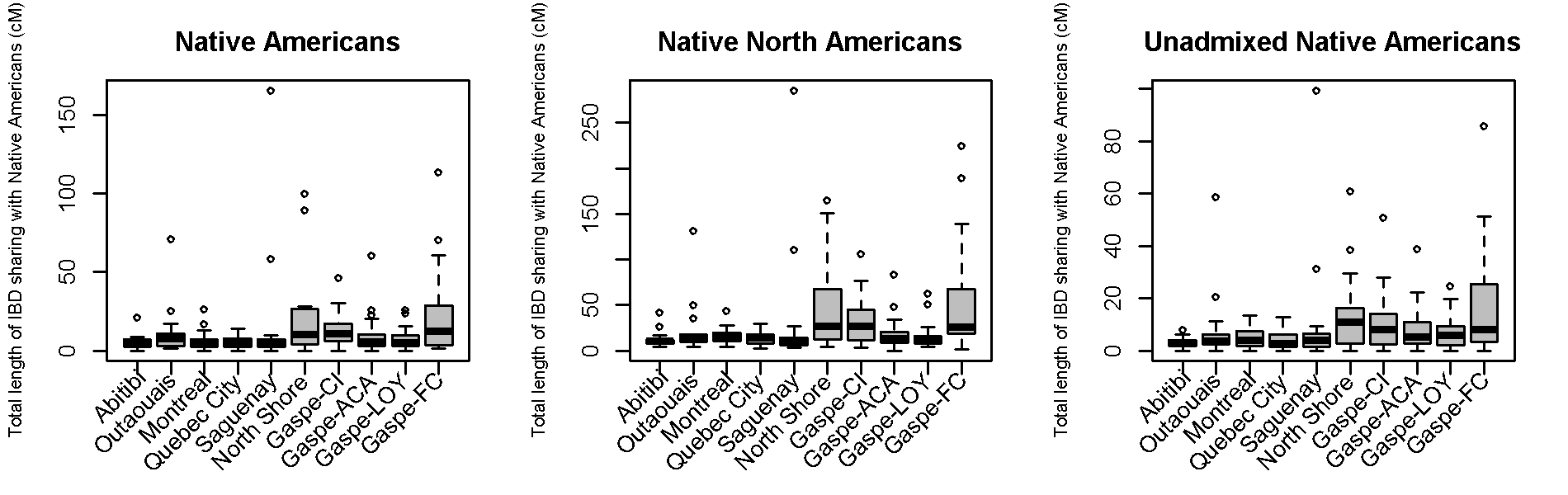

Supplement: Figure S3 — IBD sharing between the Quebec population groups and different Native American populations. Boxplots of total length of IBD sharing between the Quebec individuals and different Native American reference populations from 3 runs of fastIBD. See Table S2 for the description of the Native American populations. (TIFF) [file pone.0065507.s003.tiff]

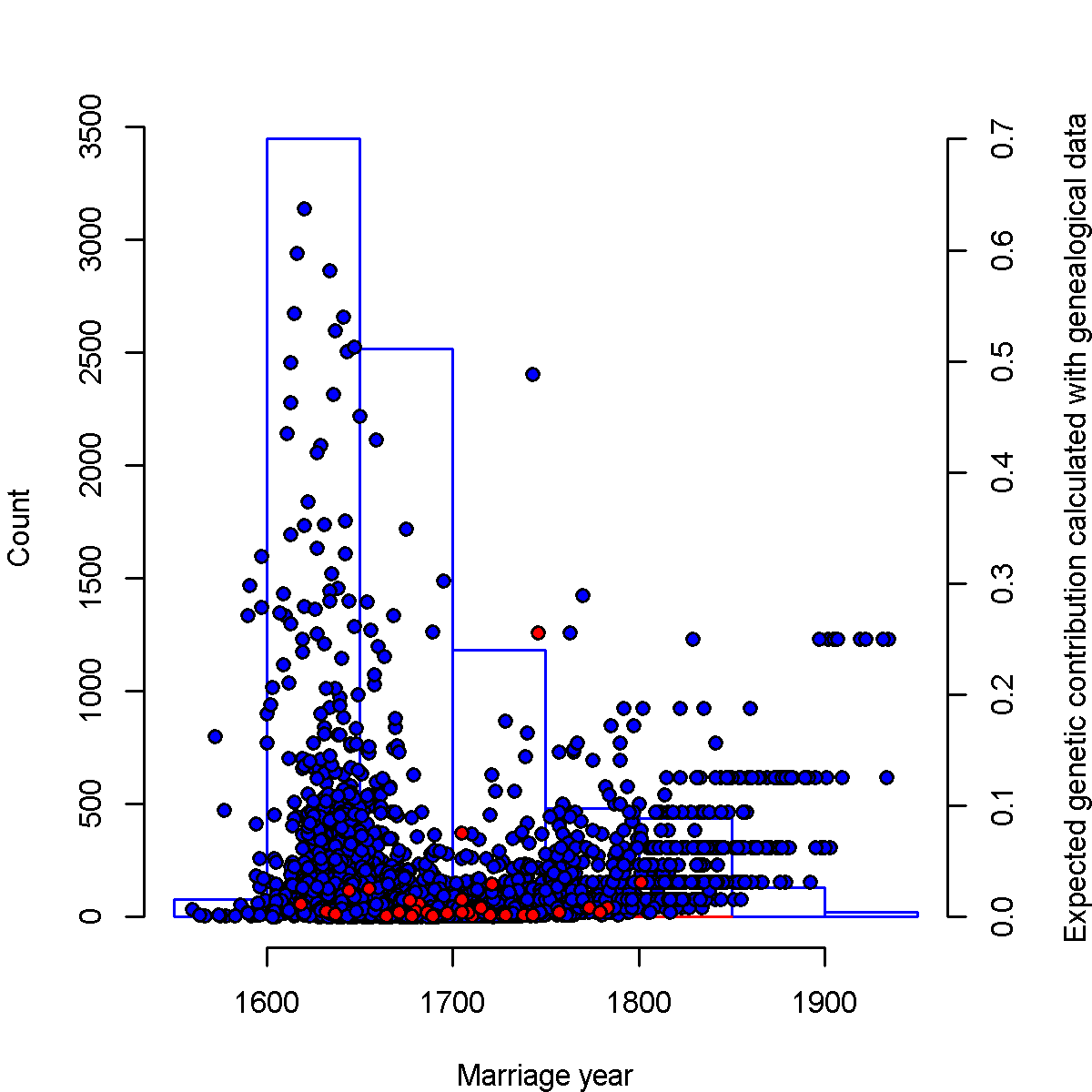

Supplement: Figure S4 — Plot of the number (bars) and genealogical genetic contribution (dots) of the Native American (red) and non Native American (blue) founders by marriage year in the genealogies of the Quebec individuals. (TIFF) [file pone.0065507.s004.tiff]

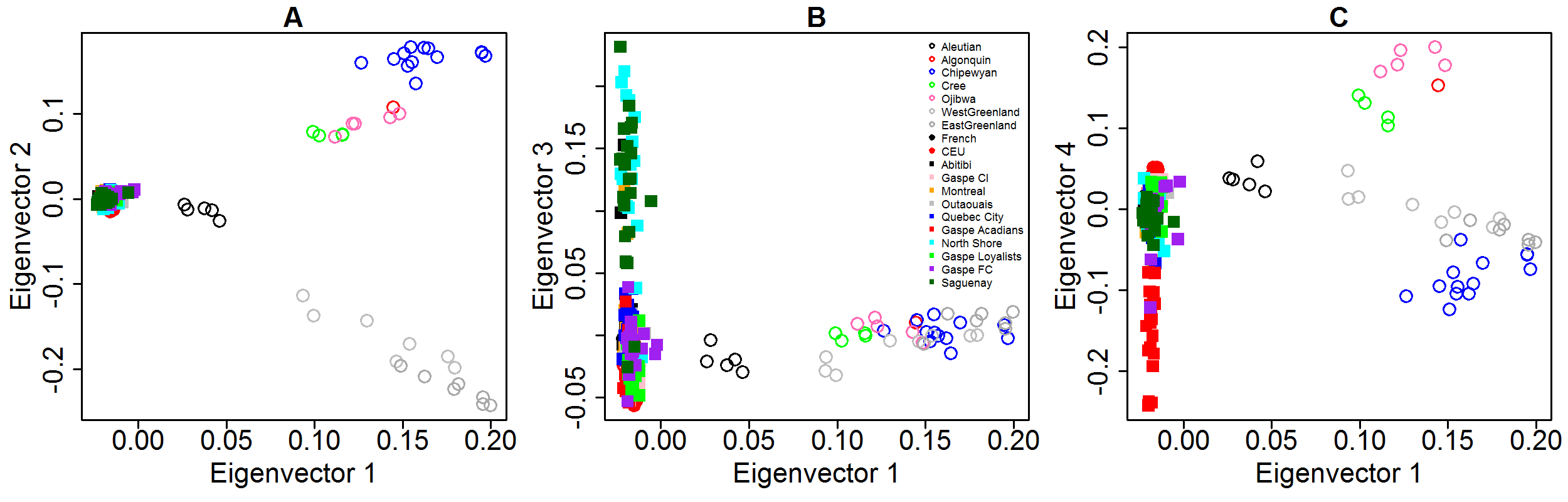

Supplement: Figure S5 — First 4 eigenvectors of the Principal Component Analysis of the genomic data. Note - Global ancestry was estimated by PCA on the genotypic data using the EIGENSOFT software version 3.0 [40], [62]. To remove the effect of LD on the PCA, we used the subset of pruned SNPs described above. (TIFF) [file pone.0065507.s005.tiff]
