# Supplementary material for: Native American Admixture in the Quebec Founder Population
Source: PLoS One. 2013 Jun 12;8(6):e65507. doi: 10.1371/journal.pone.0065507 (PMC3680396; doi:10.1371/journal.pone.0065507)
Supplement: Table S1 — Genetic contribution and admixture indices among Quebec individuals. (DOCX) [file pone.0065507.s006.docx]

**Table S1. Genetic contribution and admixture indices among Quebec individuals.**

| **Sample code** | **Expected GC of Native American founders (%)** | **Native American ancestry (ADMIXTURE) (%)** | **Native American SNVs (HAPMIX) (%)** | **Total length of shared IBD with Native Americans**  **(cM)** | **mtDNA HG** | **Y chromosome HG** |
| --- | --- | --- | --- | --- | --- | --- |
| abi002 | 0.05 | 3.11 | 0.77 | 9.5 | H | NA |
| abi003 | 0.02 | 1.83 | 0.37 | 8.0 | H | NA |
| abi004 | 1.81 | 1.20 | 1.45 | 17.0 | K | NA |
| abi005 | 0.05 | 0.02 | 0.47 | 7.6 | T2b | NA |
| abi006 | 0.00 | 2.42 | 1.22 | 4.2 | D | NA |
| abi008 | 0.00 | 0.12 | 0.69 | 3.8 | H | NA |
| abi009 | 0.00 | 2.35 | 0.19 | 14.7 | T2b | NA |
| abi010 | 0.02 | 0.00 | 0.48 | 11.0 | V | NA |
| abi014 | 0.20 | 1.21 | 1.09 | 26.0 | U5a1 | NA |
| abi017 | 0.20 | 0.00 | 1.55 | 10.3 | I | NA |
| abi018 | 0.05 | 0.10 | 0.98 | 11.1 | H | NA |
| abi019 | 0.25 | 2.20 | 0.32 | 11.1 | U5a1 | NA |
| abi023 | 0.00 | 1.42 | 0.48 | 11.6 | H | NA |
| abi024 | 0.31 | 2.21 | 1.57 | 41.3 | U3 | NA |
| abi025 | 0.05 | 1.93 | 1.01 | 7.3 | H | NA |
| abi026 | 0.24 | 3.04 | 0.60 | 9.0 | V | NA |
| abi027 | 0.06 | 0.00 | 1.50 | 11.6 | H | NA |
| abi030 | 0.00 | 0.74 | 0.33 | 3.7 | K | NA |
| aca001 | 0.29 | 4.20 | 1.25 | 17.8 | H1 | R1(xR1a) |
| aca002 | 0.00 | 1.19 | 0.95 | 9.3 | H | R1(xR1a) |
| aca003 | 0.05 | 0.05 | 1.09 | 21.0 | HV | R1(xR1a) |
| aca004 | 0.00 | 1.92 | 0.26 | 8.6 | H1 | R1(xR1a) |
| aca005 | 0.00 | 1.88 | 0.32 | 8.7 | T | R1(xR1a) |
| aca006 | 0.00 | 1.82 | 0.24 | 13.7 | H1 | R1(xR1a) |
| aca007 | 0.00 | 2.31 | 1.31 | 10.6 | W | R1(xR1a) |
| aca008 | 0.10 | 1.63 | 0.69 | 7.4 | H1 | R1(xR1a) |
| aca009 | 0.20 | 1.64 | 2.22 | 19.1 | H1 | R1(xR1a) |
| aca010 | 0.00 | 1.39 | 0.49 | 8.2 | T2b | I |
| aca012 | 0.05 | 1.96 | 1.51 | 14.5 | H3 | R1(xR1a) |
| aca013 | 0.10 | 0.92 | 1.81 | 47.8 | H | R1(xR1a) |
| aca014 | 0.00 | 1.87 | 0.24 | 10.5 | H1 | R1(xR1a) |
| aca015 | 0.05 | 2.40 | 0.71 | 5.8 | NA | R1(xR1a) |
| aca016 | 0.49 | 1.69 | 3.51 | 83.2 | H | R1(xR1a) |
| aca017 | 0.05 | 1.46 | 1.01 | 11.8 | H1 | R1(xR1a) |
| aca018 | 0.00 | 0.94 | 1.61 | 0.0 | H | R1(xR1a) |
| aca019 | 0.44 | 3.30 | 0.62 | 17.0 | W | R1(xR1a) |
| aca020 | 0.00 | 0.89 | 1.19 | 27.1 | H1 | R1(xR1a) |
| aca021 | 0.24 | 2.00 | 0.15 | 34.1 | H | R1(xR1a) |
| caf002 | 0.78 | 1.40 | 1.09 | 19.1 | U5a1 | R1(xR1a) |
| caf003 | 0.05 | 1.63 | 0.56 | 21.6 | H | R1(xR1a) |
| caf004 | 0.21 | 3.83 | 1.32 | 20.4 | H | R1(xR1a) |
| caf005 | 2.34 | 1.78 | 4.23 | 97.6 | H1 | R1(xR1a) |
| caf006 | 0.78 | 0.94 | 1.49 | 13.2 | H1 | J2 |
| caf007 | 0.10 | 1.27 | 0.34 | 17.6 | H | I |
| caf008 | 0.20 | 2.59 | 1.75 | 26.5 | H | J2 |
| caf009 | 0.15 | 0.21 | 0.31 | 1.8 | H1 | R1(xR1a) |
| caf010 | 0.39 | 1.30 | 1.39 | 29.1 | I | R1(xR1a) |
| caf011 | 1.22 | 2.78 | 2.25 | 30.1 | V | R1(xR1a) |
| caf012 | 1.61 | 1.38 | 0.97 | 24.5 | T2b | I |
| caf014 | 1.37 | 5.28 | 3.35 | 117.3 | J1 | R1(xR1a) |
| caf015 | 3.32 | 5.53 | 6.94 | 139.0 | H1 | R1(xR1a) |
| caf016 | 0.10 | 2.29 | 1.03 | 4.1 | T2b | R1(xR1a) |
| caf017 | 0.10 | 0.92 | 1.01 | 21.9 | H1 | R1(xR1a) |
| caf018 | 0.22 | 1.03 | 1.74 | 14.8 | T | I |
| caf019 | 5.20 | 6.76 | 5.15 | 188.8 | C1 | R1(xR1a) |
| caf020 | 4.59 | 8.60 | 10.72 | 224.1 | C1 | R1(xR1a) |
| caf021 | 0.10 | 4.01 | 2.82 | 33.7 | U5b | R1(xR1a) |
| caf022 | 0.05 | 2.64 | 1.52 | 37.7 | H1 | J2 |
| cno001 | 0.05 | 1.07 | 0.57 | 9.0 | H | R1(xR1a) |
| cno002 | 0.10 | 3.72 | 5.56 | 111.3 | K | R1(xR1a) |
| cno003 | 0.15 | 4.24 | 2.16 | 40.2 | T1 | R1a1 |
| cno004 | 0.02 | 0.00 | 0.72 | 4.1 | I | R1(xR1a) |
| cno005 | 0.05 | 0.73 | 1.53 | 13.4 | T2b | E |
| cno006 | 0.88 | 2.22 | 2.35 | 49.8 | T2b | E |
| cno007 | 0.00 | 1.56 | 0.72 | 22.3 | I | R1(xR1a) |
| cno008 | 1.66 | 5.44 | 6.05 | 164.1 | U6 | R1(xR1a) |
| cno009 | 0.00 | 3.13 | 2.69 | 28.6 | H5 | R1(xR1a) |
| cno010 | 0.27 | 2.46 | 0.98 | 42.7 | NA | R1(xR1a) |
| cno011 | 1.64 | 2.85 | 3.37 | 81.3 | H | J2 |
| cno012 | 0.00 | 0.71 | 0.90 | 11.1 | HV | NA |
| cno013 | 1.32 | 3.93 | 1.91 | 25.3 | I | R1(xR1a) |
| cno014 | 0.18 | 2.18 | 1.57 | 65.6 | H | R1(xR1a) |
| cno015 | 0.20 | 3.87 | 3.02 | 69.7 | W | R1(xR1a) |
| cno016 | 0.15 | 2.25 | 1.00 | 10.4 | T2b | R1(xR1a) |
| cno017 | 0.05 | 1.22 | 0.80 | 14.4 | K | R1(xR1a) |
| cno018 | 0.12 | 0.00 | 0.45 | 7.7 | I | R1(xR1a) |
| cno019 | 1.17 | 3.84 | 3.97 | 150.7 | K | R1(xR1a) |
| cno020 | 0.32 | 2.95 | 0.75 | 14.3 | I | R1(xR1a) |
| ian001 | 0.00 | 3.16 | 0.55 | 15.1 | J | R1(xR1a) |
| ian002 | 0.00 | 2.13 | 0.35 | 8.4 | U5b | R1(xR1a) |
| ian003 | 0.00 | 2.66 | 1.32 | 6.7 | H | R1(xR1a) |
| ian005 | 0.00 | 3.28 | 0.65 | 2.9 | H1 | I |
| ian007 | 0.00 | 3.31 | 0.32 | 5.8 | H5 | R1(xR1a) |
| ian008 | 0.39 | 0.59 | 2.66 | 50.2 | H1 | R1(xR1a) |
| ian009 | 2.44 | 0.99 | 1.07 | 29.7 | J1b | R1(xR1a) |
| ian010 | 0.78 | 3.87 | 1.05 | 39.8 | T2b | R1(xR1a) |
| ian011 | 0.00 | 1.83 | 0.09 | 8.2 | H | NA |
| ian012 | 1.56 | 2.62 | 1.36 | 19.4 | J1b | E |
| ian013 | 0.00 | 1.58 | 0.30 | 15.7 | H | I |
| ian014 | 2.78 | 1.18 | 0.85 | 26.5 | H | R1(xR1a) |
| ian015 | 0.59 | 2.11 | 1.75 | 40.0 | H1 | R1(xR1a) |
| ian016 | 0.20 | 6.07 | 1.67 | 24.6 | T | R1(xR1a) |
| ian017 | 0.00 | 3.76 | 3.40 | 67.4 | J1 | R1(xR1a) |
| ian018 | 1.86 | 3.66 | 3.87 | 105.2 | K | R1(xR1a) |
| ian019 | 0.61 | 2.06 | 0.43 | 26.5 | K | R1(xR1a) |
| ian020 | 0.78 | 2.35 | 2.64 | 59.8 | C1 | I |
| ian021 | 2.56 | 5.43 | 2.36 | 76.9 | C1 | I |
| ian022 | 1.76 | 2.77 | 0.86 | 37.7 | C1 | R1(xR1a) |
| loy001 | 0.00 | 2.80 | 0.38 | 8.1 | T | I |
| loy002 | 0.00 | 1.23 | 1.11 | 15.2 | K | R1(xR1a) |
| loy003 | 0.00 | 4.97 | 1.05 | 4.4 | H | R1(xR1a) |
| loy004 | 0.00 | 3.75 | 0.72 | 12.4 | J1a | R1(xR1a) |
| loy005 | 0.00 | 2.55 | 0.58 | 6.8 | H1 | R1a1 |
| loy006 | 0.00 | 4.50 | 0.45 | 6.1 | HV | R1(xR1a) |
| loy007 | 0.00 | 2.46 | 0.79 | 15.7 | U2 | I |
| loy008 | 0.78 | 2.47 | 1.21 | 8.3 | J | R1(xR1a) |
| loy009 | 0.78 | 3.89 | 1.56 | 61.7 | H | R1(xR1a) |
| loy010 | 0.10 | 1.23 | 1.23 | 24.3 | H | I |
| loy012 | 0.00 | 1.98 | 0.26 | 9.3 | H1 | R1(xR1a) |
| loy013 | 0.00 | 1.14 | 0.69 | 7.0 | H | F(xI,J2,K) |
| loy014 | 0.98 | 3.70 | 2.06 | 50.7 | HV | R1(xR1a) |
| loy015 | 0.00 | 3.48 | 1.50 | 25.5 | H | I |
| loy016 | 0.00 | 3.17 | 0.67 | 7.9 | I | F(xI,J2,K) |
| loy017 | 0.00 | 3.88 | 0.97 | 9.4 | K | R1(xR1a) |
| loy018 | 0.00 | 1.86 | 1.11 | 7.6 | J1 | R1a1 |
| loy019 | 0.00 | 1.61 | 0.36 | 11.4 | U2 | R1(xR1a) |
| loy020 | 0.20 | 1.77 | 0.50 | 12.7 | J | R1(xR1a) |
| loy021 | 0.00 | 1.58 | 0.26 | 16.4 | K | R1(xR1a) |
| mon001 | 0.10 | 1.91 | 1.19 | 19.5 | U5a1 | F(xI,J2,K) |
| mon002 | 0.42 | 2.77 | 0.54 | 11.0 | X | J2 |
| mon003 | NA | 0.04 | 0.47 | 6.2 | H | R1(xR1a) |
| mon004 | 0.17 | 0.97 | 1.76 | 22.2 | K | I |
| mon005 | 0.10 | 3.65 | 1.24 | 15.8 | H5 | I |
| mon006 | 0.07 | 2.74 | 1.17 | 43.0 | H | E |
| mon007 | 0.00 | 2.66 | 1.49 | 8.7 | H1 | R1(xR1a) |
| mon008 | 0.16 | 2.62 | 0.89 | 20.9 | H1 | R1a1 |
| mon009 | 0.07 | 1.94 | 0.89 | 14.7 | T1 | I |
| mon010 | 0.00 | 1.80 | 0.32 | 9.3 | J1a | R1(xR1a) |
| mon011 | 0.00 | 0.00 | 0.62 | 11.6 | H5 | R1(xR1a) |
| mon012 | 0.10 | 2.54 | 0.33 | 10.1 | U | J2 |
| mon013 | 0.09 | 1.66 | 0.74 | 18.8 | K | NA |
| mon014 | 0.11 | 2.12 | 1.68 | 19.1 | N1b | R1(xR1a) |
| mon015 | 0.06 | 1.25 | 1.81 | 17.2 | X | NA |
| mon016 | 0.39 | 0.00 | 1.92 | 23.0 | H | R1(xR1a) |
| mon017 | 0.01 | 1.93 | 1.31 | 10.1 | T | R1(xR1a) |
| mon018 | 0.05 | 1.15 | 1.77 | 27.3 | H | NA |
| mon019 | 0.00 | 2.20 | 0.26 | 9.3 | H | NA |
| mon020 | NA | 3.01 | 0.95 | 27.9 | H5 | I |
| mon021 | 0.15 | 2.52 | 0.43 | 17.8 | U5a1 | NA |
| mon022 | NA | 1.40 | 0.58 | 4.3 | U5a1 | R1(xR1a) |
| mon225 | NA | 0.98 | 1.93 | 13.7 | K | NA |
| mon259 | 0.39 | 0.66 | 1.12 | 19.6 | H | R1(xR1a) |
| mon510 | NA | 1.57 | 1.89 | 7.6 | K | NA |
| out001 | 0.15 | 2.50 | 0.76 | 6.1 | T2b | NA |
| out002 | 1.59 | 3.38 | 1.27 | 34.8 | H | NA |
| out006 | 0.27 | 0.00 | 1.01 | 14.6 | W | NA |
| out008 | 0.00 | 2.31 | 0.32 | 17.3 | H | NA |
| out009 | 0.00 | 1.57 | 0.78 | 10.2 | W | NA |
| out010 | 0.49 | 3.99 | 1.83 | 49.4 | K | NA |
| out014 | 0.00 | 2.01 | 0.58 | 17.2 | UK | NA |
| out025 | 0.04 | 4.54 | 4.13 | 131.2 | H | NA |
| out101 | 0.05 | 0.92 | 0.56 | 11.5 | K | NA |
| out102 | 0.00 | 0.47 | 0.79 | 7.7 | K | NA |
| out103 | 0.44 | 2.48 | 2.04 | 12.4 | H | NA |
| out104 | NA | 0.62 | 1.75 | 11.4 | H | NA |
| out105 | 0.00 | 0.00 | 0.35 | 8.4 | UK | NA |
| out106 | 0.00 | 3.17 | 0.98 | 4.1 | I | NA |
| out107 | 0.37 | 1.19 | 0.65 | 17.2 | T1 | NA |
| que001 | 0.20 | 2.21 | 1.49 | 7.5 | T1 | NA |
| que002 | 0.10 | 2.08 | 0.56 | 2.0 | K | NA |
| que005 | 0.00 | 1.14 | 0.38 | 17.0 | J1b1 | NA |
| que008 | 0.00 | 1.55 | 0.87 | 14.5 | V | NA |
| que009 | 0.15 | 1.04 | 0.56 | 11.5 | H | NA |
| que010 | 0.32 | 1.96 | 1.44 | 26.0 | K | NA |
| que011 | 0.00 | 2.72 | 2.63 | 5.7 | H | NA |
| que012 | 0.20 | 1.95 | 0.65 | 8.1 | H | NA |
| que015 | 0.22 | 1.69 | 1.29 | 17.5 | D | NA |
| que017 | 0.04 | 2.94 | 0.54 | 18.3 | T2b | NA |
| que018 | 0.29 | 0.91 | 1.74 | 8.0 | H | NA |
| que019 | 0.90 | 1.23 | 0.47 | 4.4 | I | NA |
| que021 | 0.23 | 1.70 | 0.79 | 29.9 | T2b | NA |
| que022 | 0.00 | 1.94 | 0.11 | 14.9 | H | NA |
| que024 | NA | 3.15 | 0.30 | 10.0 | NA | NA |
| que027 | 0.02 | 0.37 | 0.76 | 9.7 | H | NA |
| que034 | 0.00 | 1.83 | 0.76 | 22.3 | H | NA |
| que039 | NA | 0.12 | 1.02 | 6.9 | NA | NA |
| que042 | 0.00 | 2.92 | 0.96 | 10.8 | H | NA |
| que056 | 0.05 | 0.75 | 1.43 | 16.2 | H | NA |
| que066 | 0.54 | 1.24 | 0.25 | 15.1 | H | NA |
| que070 | 0.17 | 2.00 | 1.71 | 19.0 | K | NA |
| que071 | 0.02 | 3.81 | 0.23 | 9.8 | B | NA |
| que077 | 0.12 | 3.31 | 2.02 | 24.8 | J1a | NA |
| que085 | NA | 0.00 | 0.92 | 16.5 | NA | NA |
| sag001 | 0.29 | 0.50 | 1.10 | 5.7 | NA | NA |
| sag002 | 0.16 | 3.23 | 1.60 | 26.8 | NA | NA |
| sag003 | 0.05 | 1.18 | 0.28 | 5.3 | H3 | R1(xR1a) |
| sag004 | 0.15 | 1.35 | 1.15 | 3.1 | H1 | R1(xR1a) |
| sag005 | 0.00 | 0.38 | 0.53 | 8.8 | NA | NA |
| sag006 | 0.02 | 0.61 | 1.01 | 2.9 | NA | NA |
| sag007 | 0.23 | 2.57 | 1.27 | 16.5 | NA | NA |
| sag008 | 0.11 | 2.29 | 0.44 | 13.3 | NA | NA |
| sag009 | 0.20 | 3.03 | 0.36 | 14.4 | NA | NA |
| sag010 | 0.02 | 0.38 | 0.74 | 5.3 | NA | NA |
| sag011 | 0.31 | 1.15 | 1.21 | 22.2 | NA | NA |
| sag013 | 0.00 | 1.50 | 1.27 | 12.3 | T2b | I |
| sag014 | 0.57 | 2.53 | 1.05 | 7.9 | K | R1(xR1a) |
| sag015 | 0.05 | 0.13 | 0.97 | 7.6 | NA | NA |
| sag016 | 0.34 | 1.23 | 1.40 | 8.6 | NA | NA |
| sag017 | 0.00 | 0.50 | 0.69 | 6.4 | T2b | R1(xR1a) |
| sag018 | 0.10 | 2.75 | 1.03 | 12.3 | NA | NA |
| sag019 | 0.00 | 1.16 | 0.57 | 4.3 | NA | NA |
| sag020 | 0.15 | 2.11 | 0.86 | 9.9 | NA | NA |
| sag021 | 0.61 | 7.89 | 8.31 | 285.2 | NA | NA |
| sag022 | 0.29 | 2.95 | 0.92 | 11.0 | NA | NA |
| sag023 | NA | 3.47 | 4.26 | 109.8 | NA | NA |

For each Quebec individual: Genetic contribution (GC) of the Native American founders based on genealogical data, Native American ancestry inferred by the ADMIXTURE software, percentage of SNVs coming from the Native Americans as estimated by the HAPMIX software, total length of IBD segments shared with the Native Americans, mtDNA D-loop haplogroup (HG) and the Y-chromosome haplogroup [[1](#_ENREF_1)]

1. Moreau C, Vezina H, Yotova V, Hamon R, de Knijff P, et al. (2009) Genetic heterogeneity in regional populations of Quebec--parental lineages in the Gaspe Peninsula. Am J Phys Anthropol 139: 512-522.
