# Supplementary material for: Native American Admixture in the Quebec Founder Population
Source: PLoS One. 2013 Jun 12;8(6):e65507. doi: 10.1371/journal.pone.0065507 (PMC3680396; doi:10.1371/journal.pone.0065507)
Supplement: Table S2 — Reference populations used in the analyses. (DOCX) [file pone.0065507.s007.docx]

**Table S2. Reference populations used in the analyses.**

|  | **Population** | **Number of individuals** |
| --- | --- | --- |
| **Native North Americans** | Aleutian | 8 |
|  | Algonquin | 5 |
|  | Chipewyan | 15 |
|  | Cree | 4 |
|  | Ojibwa | 5 |
|  | WestGreenland | 8 |
|  | EastGreenland | 7 |
| **Native South Americans**  **Native South Americans** | Arara | 1 |
|  | Arhuaco | 5 |
|  | Aymara | 23 |
|  | Bribri | 4 |
|  | Cabecar | 31 |
|  | Chane | 2 |
|  | Chilote | 8 |
|  | Chono | 4 |
|  | Chorotega | 1 |
|  | Diaguita | 5 |
|  | Embera | 5 |
|  | Guahibo | 6 |
|  | Guarani | 6 |
|  | Guaymi | 5 |
|  | Huetar | 1 |
|  | Hulliche | 4 |
|  | Inga | 9 |
|  | Jamamadi | 1 |
|  | Kaingang | 2 |
|  | Kaqchikel | 13 |
|  | Karitiana | 13 |
|  | Kogi | 4 |
|  | Maleku | 3 |
|  | Maya1 | 37 |
|  | Maya2 | 12 |
|  | Mixe | 17 |
|  | Mixtec | 5 |
|  | Palikur | 3 |
|  | Parakana | 1 |
|  | Piapoco | 7 |
|  | Pima | 33 |
|  | Purepecha | 1 |
|  | Quechua | 40 |
|  | Surui | 24 |
|  | Teribe | 3 |
|  | Ticuna | 6 |
|  | Toba | 4 |
|  | Waunana | 3 |
|  | Wayuu | 11 |
|  | Wichi | 5 |
|  | Yaghan | 4 |
|  | Yaqui | 1 |
|  | Zapotec1 | 22 |
|  | Zapotec2 | 21 |
|  | Tepehuano | 25 |
| **Europeans** | CEU | 108 |
|  | French | 28 |
|  | Italian | 12 |
|  | TSI | 88 |
|  | Tuscan | 8 |
| **Siberians** | Altaian | 12 |
|  | Buryat | 17 |
|  | Chukchi | 30 |
|  | Dolgan | 4 |
|  | Evenki | 15 |
|  | Ket | 2 |
|  | Khanty | 35 |
|  | Koryak | 10 |
|  | Mongolian | 8 |
|  | Naukan | 16 |
|  | Nganasan1 | 8 |
|  | Nganasan2 | 14 |
|  | Selkup | 9 |
|  | Tundra_Nentsi | 3 |
|  | Tuvinians | 15 |
|  | Yakut | 34 |
|  | Yukaghir | 13 |

This genotype data was described in [[1](#_ENREF_1)]. The masked dataset, excluding Native American’s genomic regions of European or African origin, was used in our analyses whenever possible (ADMIXtools, ALDER and IBD sharing analyses). For the analyses requiring phased data (ADMIXTURE, HAPMIX and principal components analysis (PCA)), the unmasked dataset, including all genomic regions of the Native American populations, was used. Retaining only markers overlapping in all datasets yielded to a total of 196,722 SNVs.

163 unadmixed Native Americans (devoid of segments of European or African origin) have been selected by Reich et al., from which 5 were from North America and 158 from South America.

1. Reich D, Patterson N, Campbell D, Tandon A, Mazieres S, et al. (2012) Reconstructing Native American population history. Nature 488: 370-374.
