# Supplementary material for: Native American Admixture in the Quebec Founder Population
Source: PLoS One. 2013 Jun 12;8(6):e65507. doi: 10.1371/journal.pone.0065507 (PMC3680396; doi:10.1371/journal.pone.0065507)
Supplement: Table S3 — Genetic contribution and admixture indices in the Quebec subpopulations. (DOCX) [file pone.0065507.s008.docx]

**Table S3. Genetic contribution and admixture indices in the Quebec subpopulations.**

| **Regional/ethnocultural population group** | **Expected genetic contribution of Native Americans**  **(genealogical data)^a^** | | **Native American ancestry (ADMIXTURE)^b^** | | **Native American SNVs**  **(HAPMIX)^b^** | | **Total length of haplotypes shared with Native Americans**  **(fastIBD)^a^** | |
| --- | --- | --- | --- | --- | --- | --- | --- | --- |
| **(n genotypes; n genealogies)** | **Median (%)** | **IQR** | **Median (%)** | **IQR** | **Median (%)** | **IQR** | **Median (cM)** | **IQR** |
| Abitibi (18;18) | 0.05 | 0.19 | 1.31 | 2.10 | 0.73 | 0.72 | 11 | 4 |
| Outaouais (15;14) | 0.05 | 0.34 | 2.01 | 2.07 | 0.79 | 0.89 | 12 | 8 |
| Montreal (25;20) | 0.09 | 0.11 | 1.91 | 1.40 | 1.12 | 1.11 | 16 | 10 |
| Quebec City (25;22) | 0.11 | 0.21 | 1.83 | 1.07 | 0.79 | 0.88 | 14 | 9 |
| Saguenay (22;21) | 0.15 | 0.27 | 1.42 | 1.96 | 1.02 | 0.55 | 9 | 8 |
| North Shore (20;20) | 0.15 | 0.41 | 2.36 | 2.56 | 1.55 | 1.99 | 27 | 54 |
| Gaspesian Channel Islanders (20;20) | 0.49 | 1.61 | 2.64 | 1.39 | 1.06 | 1.38 | 27 | 29 |
| Gaspesian Acadians (20;20) | 0.05 | 0.12 | 1.75 | 0.63 | 0.98 | 0.91 | 13 | 11 |
| Gaspesian Loyalists (20;20) | 0.00 | 0.02 | 2.51 | 1.99 | 0.75 | 0.65 | 10 | 8 |
| Gaspesian French Canadians (20;20) | 0.31 | 1.33 | 2.03 | 2.58 | 1.51 | 1.92 | 25 | 34 |
| **Total sample** (205;195) | **0.10** | **0.29** | **1.94** | **1.62** | **1.01** | **0.99** | **14** | **17** |

Here Quebec regions are presented with (number of individuals with genotype data; number of individuals with genealogical data). For each population, Native American genetic contribution was estimated using genealogical data. The Native American ancestry proportions in the Quebec subpopulations was estimated with 1) the ADMIXTURE software [[1](#_ENREF_1)] 2) the HAPMIX software [[2](#_ENREF_2)], and 3) IBD sharing analysis using the fastIBD software [[3](#_ENREF_3)]. For more details on the analyses, see figure and table legends. For each measure, we tested for differences among subpopulations (excluding the total Quebec sample (in bold)) using a Kruskal-Wallis test. The R statistical environment version 2.15.0 Patched [[4](#_ENREF_4)] was used for the Kruskal-Wallis tests on the subpopulations.

IQR = interquartile range.

^a^ Kruskal-Wallis test p<0.001.

^b^ Kruskal-Wallis test p<0.05.

1. Alexander DH, Novembre J, Lange K (2009) Fast model-based estimation of ancestry in unrelated individuals. Genome Res 19: 1655-1664.

2. Price AL, Tandon A, Patterson N, Barnes KC, Rafaels N, et al. (2009) Sensitive detection of chromosomal segments of distinct ancestry in admixed populations. PLoS Genet 5: e1000519.

3. Browning BL, Browning SR (2011) A fast, powerful method for detecting identity by descent. Am J Hum Genet 88: 173-182.

4. Team RC (2013) R: A language and environment for

statistical computing. R Foundation for Statistical Computing. Vienna.
